# *Notes from the Field:* Diagnosis of Congenital Syphilis and Syphilis Among Females of Reproductive Age Before and During the COVID-19 Pandemic — Chicago, 2015–2022

**DOI:** 10.15585/mmwr.mm7247a2

**Published:** 2023-11-24

**Authors:** Helen E. Cejtin, Eric F. Warren, Taylor Guidry, Katherine Boss, Ashley Becht, Irina Tabidze

**Affiliations:** ^1^John H. Stroger Hospital of Cook County, Chicago, Illinois; ^2^Feinberg School of Medicine, Northwestern University, Chicago, Illinois; ^3^Division of STD Prevention, National Center for HIV, Viral Hepatitis, STD, and TB Prevention, CDC; ^4^Syndemic Infectious Disease Bureau, Chicago Department of Public Health, Chicago, Illinois.

Syphilis is a bacterial infection that is of particular concern during pregnancy because of the risk for transplacental fetal infection. Pregnancies complicated by untreated syphilis are at increased risk for adverse outcomes, including stillbirth and long-term physical and cognitive sequelae in the affected infant. After implementation of enhanced efforts (*1*) by the Chicago Department of Public Health (CDPH) to eliminate congenital syphilis, including improvements to the surveillance and case management system, the number of Chicago congenital syphilis cases steadily decreased during 2015–2019, despite national increases in congenital syphilis and local increases in syphilis among females of reproductive age.* In 2020, with the emergence of the COVID-19 pandemic, the trend in Chicago abruptly shifted, and cases of congenital syphilis increased during the next 3 years.

## Investigation and Outcomes

### Data Collection and Analyses

To evaluate missed prevention opportunities and whether they differed during the prepandemic era (2015–2019) compared with the COVID-19 pandemic (2020–2022), CDPH conducted a comprehensive review of all congenital syphilis cases reported during 2015–2022, including case investigation report forms, disease intervention specialist interview records and field notes, and medical record abstraction (when available). Missed prevention opportunities were categorized into one of the following five mutually exclusive categories: 1) no adequate maternal treatment despite receipt of a timely syphilis diagnosis, 2) no timely prenatal care and no timely syphilis testing, 3) late identification of seroconversion during pregnancy, 4) no timely syphilis testing despite receipt of timely prenatal care, and 5) clinical evidence of syphilis despite maternal completion of treatment (*2*). Missed opportunities for congenital syphilis prevention before and during the COVID-19 pandemic were compared using Pearson’s chi-square test, with p-values <0.01 considered statistically significant. This activity was reviewed by CDC and CDPH, deemed not research, and was conducted consistent with applicable federal law and CDC policy.^†^

### Outcomes

During 2020–2022, reported congenital syphilis cases in Chicago increased an average of 74.1% per year, more than 3 times the increase in the rate of reported cases of syphilis in females of reproductive age (22.1% per year) during the same period (Table). The rate of reported syphilis in females of reproductive age decreased 9.5% in 2022 compared with 2021. During 2015–2019, among 67 congenital syphilis cases, 18 (26.9%) resulted from inadequate maternal treatment despite timely syphilis diagnoses; during 2020–2022, this percentage increased to 48.3% (43 of 89 cases), representing approximately an 80% increase (p = 0.007). During the pandemic years, this percentage increased each year, from 31.6% (six of 19) in 2020, to 40.0% (10 of 25) in 2021, and to 60.0% (27 of 45) in 2022. Among 67 congenital syphilis cases that occurred during 2015–2019, a total of 14 (20.9%) resulted from late identification of seroconversion during pregnancy. Although the percentage of such cases in 2020 was significantly higher (10 of 19; 52.6%) (p = 0.006), the overall percentage of congenital syphilis cases resulting from late identification of seroconversion during pregnancy was similar during 2020–2022 (20 of 89; 22.5%). Although the percentage of cases resulting from absence of prenatal care and timely testing decreased overall, from 40.3% (27) prepandemic to 29.2% (26) during the pandemic, the number of cases in this category in 2021 and 2022 were the highest they had been since 2015. There were no cases due to lack of timely syphilis testing despite timely prenatal care either before or during the pandemic. During 2022, syphilis diagnoses among females of reproductive age decreased. 

**TABLE:** Changes in syphilis prevalence and missed opportunities for congenital syphilis prevention — Chicago, 2015–2022

Syphilis and CS characteristic	Year, no.									
	Prepandemic, 2015–2019						Pandemic, 2020–2022			
	2015	2016	2017	2018	2019	Total 2015–2019	2020	2021	2022	Total 2020–2022
Females of reproductive age* with syphilis and CS cases [% change from previous year]										
**Total primary or secondary syphilis cases in females of reproductive age **	**52** **[NA]**	**45** **[−3.5]**	**47** **[4.4]**	**64** **[36.2]**	**79** **[23.4]**	**287 **	**122** **[54.4]**	**148** **[21.3]**	**134** **[−9.5]**	**404^†^**
**Total CS cases **	**24** **[NA]**	**12** **[**−**50.0]**	**11** **[**−**8.3]**	**11** **[—]**	**9** **[**−**18.2]**	**67 **	**19** **[111.1]**	**25** **[31.2]**	**45** **[80.0]**	**89^§^**
**Missed CS prevention opportunities (% of total)**										
No adequate maternal treatment despite receipt of timely syphilis diagnosis	6 (25.0)	1 (8.2)	4 (36.4)	3 (23.1)	4 (44.4)	**18 (26.9)**	6 (31.6)	10 (40.0)	27 (60.0)	**43 (48.3)**
No timely prenatal care and no timely syphilis testing	10 (41.7)	5 (41.7)	4 (36.4)	5 (38.5)	3 (33.3)	**27 (40.3)**	3 (15.8)	13 (52.0)	10 (22.2)	**26 (29.2)**
Late identification of seroconversion during pregnancy	5 (20.8)	2 (16.7)	2 (18.2)	3 (23.1)	2 (22.2)	**14 (20.9)**	10 (52.6)	2 (8.0)	8 (17.8)	**20 (22.5)**
No timely syphilis testing despite receipt of timely prenatal care	0	0	0	0	0	**0 (—)**	0	0	0	**0 (—)**
Clinical evidence of syphilis despite maternal treatment completion	3 (12.5)	4 (33.3)	1 (9.1)	0	0	**8 (11.9)**	0	0	0	**8 (9.0)**

**Abbreviations:** CS = congenital syphilis; NA = not applicable.

* Females of reproductive age are persons aged 15–44 years and assigned female sex at birth.

^†^ During 2020–2022, reported syphilis cases in females of reproductive age in Chicago increased an average of 22.1% per year.

^§^ During 2020–2022, reported congenital syphilis cases in Chicago increased an average of 74.1% per year.

## Preliminary Conclusions and Actions

These data suggest that in Chicago, the pandemic-associated increase in the number of congenital syphilis cases was likely not caused solely by an increase in cases of syphilis in females of reproductive age, and that the relative contribution of the different missed opportunities changed. The increase in late identification of seroconversion during pregnancy that occurred only in 2020 could be explained by a decrease in testing for and treatment of syphilis because of pandemic-associated declines in clinic visits and closures of sexually transmitted infection clinics, as well as increased use of telemedicine rather than in-person prenatal care, which precludes the use of phlebotomy. The sustained increase in inadequate maternal treatment of diagnosed syphilis during the pandemic might be related, at least in part, to the diversion of public health resources to COVID-19 mitigation efforts, resulting in increasing challenges to contacting pregnant patients and ensuring treatment. This increase in inadequate maternal syphilis treatment might have resulted in a disproportionate increase in congenital syphilis cases relative to the more modest increase in syphilis cases among females of reproductive age. The decrease in syphilis diagnosis among females of reproductive age during 2022 could represent an actual reduction in cases resulting from improved testing and treatment services, or a decrease in diagnosis and underreporting of cases.

COVID-19 remains an ongoing public health challenge, despite the expiration of the U.S. public health emergency declaration (*3*). Efforts that support timely identification and appropriate clinical and public health management of syphilis in females of reproductive age and congenital syphilis could help reclaim the progress previously made by CDPH towards elimination of congenital syphilis. The use of provider education about congenital syphilis; electronic reporting systems; improved pregnancy ascertainment; enhanced case management for syphilis cases in pregnancy; strong partnerships with providers, community-based organizations, and maternal and child health programs; and statewide review of all congenital syphilis cases by a multidisciplinary review board that were used before the pandemic are more important now than ever in light of the rise in cases of congenital syphilis.

## Footnotes

* Females of reproductive age are persons aged 15–44 years and assigned female sex at birth.

† 45 C.F.R. part 46.102(l)(2), 21 C.F.R. part 56; 42 U.S.C. Sect. 241(d); 5 U.S.C. Sect. 552a; 44 U.S.C. Sect. 3501 et seq.
